# The Dispensatory of the United States of America

**Published:** 1843-09-02

**Authors:** 


*The Dispensatory of the United States of America.* By George B. Wood, M. D., Professor of Materia Medica and Pharmacy in the University of Pennsylvania, &c. &c., and Franklin Bache, M. D., Professor of Chemistry in Jefferson Medical College of Philadelphia. Fifth Edition, enlarged and revised. Published by Grigg & Elliott, 1843. 8vo. pp. 1360.

This is the fifth edition of our great national work. In it the indefatigable authors have fully redeemed the statement of their preface—that they have made such additions as were calculated to "maintain it on a level with the advancing knowledge of the times." They state that "in no revision of the Dispensatory have they bestowed so much labour as on the present." The new editions of our own and of the Edinburgh Pharmacopœias, as well as the numerous treatises on Materia Medica which have recently appeared in Great Britain, required necessarily many alterations, and considerable additions. "It has been the aim of the authors, by pruning redundancies and concentrating the new matter within the smallest possible space, to swell the Dispensatory as little as consisted with the great object of utility; but with all their endeavours, they have been compelled to exceed their former limits by more than one hundred pages." The work is now admirable in all the various departments of Materia Medica and Pharmacy. To it, as a text book for the medical student, it perhaps may be objected that it is rather imperfect in the Therapeutic portion; but it should be remembered that a work of this kind is limited and strictly practical in its character, and that abstract, theoretical disquisitions are misplaced. The therapeutic summary attached to each article is, though very much condensed, a judicious exposition of the medical properties of the drug.

We know of no work in which there is more industry, care, ability, and judgment shown than in the present one, and the universal opinion of the profession has ratified this estimate. We shall now briefly notice some of the new articles which have been added.

Solution of Hydriodate of Arsenic and Mercury. *Liquor Hydriodatis Arsenici et Hydrargyri. Liquor Hydrargyri et Arsenici Iodidi.* This preparation was introduced by Mr. Donovan, a chemist of Dublin, and is supposed to combine the virtues of its three ingredients. We have already published its formula, with the combined testimony of a large number of the eminent practitioners of Dublin in its favour. (p. 80.) It is recommended in various cutaneous affections, in secondary syphilis, and in some uterine disorders. The dose is 20 minims three times a day in distilled water. This quantity contains the twenty-fourth of a grain of deutoxide of mercury, and about the quarter of a grain of iodine. Dr. E. J. Taylor (*Am. Journ. Med. Sciences*, N. S. Vol. v., p. 319) states that he rarely gave more than five minims (four drops.) It sometimes produces unpleasant sensations in the head, stomach, and bowels. When these occur the medicine should be laid aside for a week or so. In some instances the solution diluted with an equal bulk of water, was used as an external application to ulcers and cutaneous eruptions, at the same time that it was administered internally. We have employed it in some cases of chronic rheumatism and secondary syphilitic disease, and with marked benefit, especially in the former affection. One case of rheumatism, apparently unconnected with any syphilitic taint, and which had resisted all the ordinary forms of treatment, disappeared soon after the exhibition of this preparation.

Sulphate of Alumina—*Aluminæ Sulphas.* This powerful antiseptic and disinfectant is prepared by saturating dilute sulphuric acid with hydrated alumina. M. Gannal first introduced it into use as a preservative of bodies. By injecting the solution (a pound avoirdupois of the salt to a quart of water) into the bloodvessels, bodies were preserved fresh in summer for twenty days or more, and in winter for three months. Cloths wetted with the solution, and laid on the body after death, will preserve it for some days in a perfectly natural state; and is in every respect preferable to ice. No notice is taken by our authors of its therapeutic properties, which are familiar to our readers from the publication of Professor Dunglison in the present volume of this journal (p. 162.) We have ourselves in some cases of chronic ulcer derived unequivocal advantage from its use. To the anatomist and pathologist it is invaluable. A solution of medium strength will be found to disinfect the hands from all unpleasant odour after being engaged in autopsies or dissections.

Oxide of Silver—*Argenti Oxidum.* This preparation is said to possess all the therapeutic virtues of the nitrate when internally exhibited, without the risk of discolouring the skin. Mr. Lane, who has recently revived its use, recommends it in the various affections of the stomach, unconnected with organic lesion, dysentery, diarrhœa, night sweats without any obvious affection, various uterine derangements, &c. &c. The dose is half a grain, given in pill twice or thrice a day. In no instance did Mr. Lane carry the dose above six grains in the twenty-four hours.

Lactate of Iron—*Ferri Lactas. Lactate of Protoxide of Iron.* On the belief that the lactic is the chief acid in the gastric juice, and that the ferruginous preparations are dissolved by it in the stomach, it was supposed that the lactate of iron ready formed, would be a useful preparation. Many French physicians of great eminence have reported strongly in its favour. Its effect in increasing the appetite is very marked. It was used in Paris principally in chlorosis, and Andral, Bouillaud, Fouquier, &c., state with great success. The dose is from one to two grains, repeated at intervals, and gradually increased. As much as twenty grains may be given in the day. It may be given in lozenge, pill, or syrup, and also in bread. The syrup is of a very light amber colour, and contains four grains of the salt to the fluidounce.

Iodide of Zinc—*Zinci Iodidum.* This is made by digesting zinc in small pieces, in excess, with iodine diffused in water. Dr. Ross, of Scotland, has used a solution, (10 to 20 grains to the ounce of water,) with great success in cases of enlarged tonsils. Dr. Goddard, of this city, informs us that he has used it in this troublesome affection with the happiest results. In a single instance we have employed it, and with decided benefit.

Wild Cherry Bark—*Prunus Virginiana.* It has been shown, by a highly intelligent young chemist of this city, Mr. William Procter, (Am. Journ. of Pharm. vol. x. p. 197,) that the hydrocyanic acid and volatile oil do not exist already formed in the bark, but result from the action of water on one of its constituents, amygdalin. In directing the infusion, the last edition of the Pharmacopœia directs the maceration to be prolonged to twenty four hours. The object of this is very manifest from what has just been said. The remark of the editors with regard to the extension of the period of maceration, (p. 1013) is by this revision rendered useless, and should have been been cancelled. It should, however, direct, in addition, that the bark be coarsely powdered; by this means the subsequent chemical action is rendered much more complete. A syrup of wild cherry bark has recently been extensively used in this city. It is prepared by macerating four ounces of the powdered bark with twelve fluid ounces of water for two days, putting the mixture in a displacement apparatus, returning the liquid which passes until it becomes clear, displacing with an additional quantity of water, until twelve fluid ounces of infusion are obtained, and then dissolving in this twenty-four ounces of sugar. (Am. Journ. of Pharm. vol. xiv. p. 27.) No heat is employed by this process, which was applied in the present instance, and some others at our suggestion,—and the virtues of this valuable medicine are more fully obtained than by any other method. The objection advanced to it by the author of the article, viz.: that "in order to give the requisite quantity of the medicine, so much sugar must be given at the same time, as to endanger embarrassment of the digestive organs," is, we are assured, perfectly groundless. We have employed it ourselves constantly for some time, and without once in any way disordering the stomach. It is a pleasant vehicle for other medicines—more especially for some of the ferruginous preparations.

We shall continue our notice in a succeeding number.

